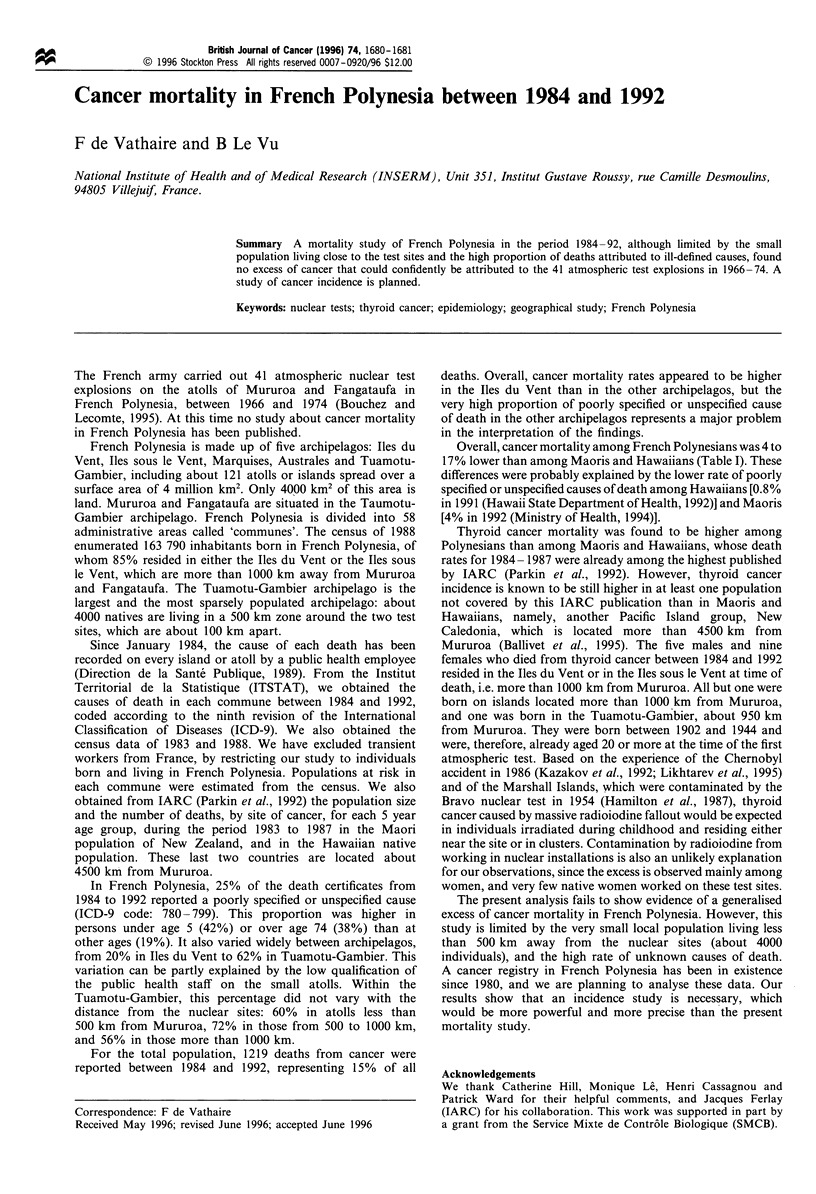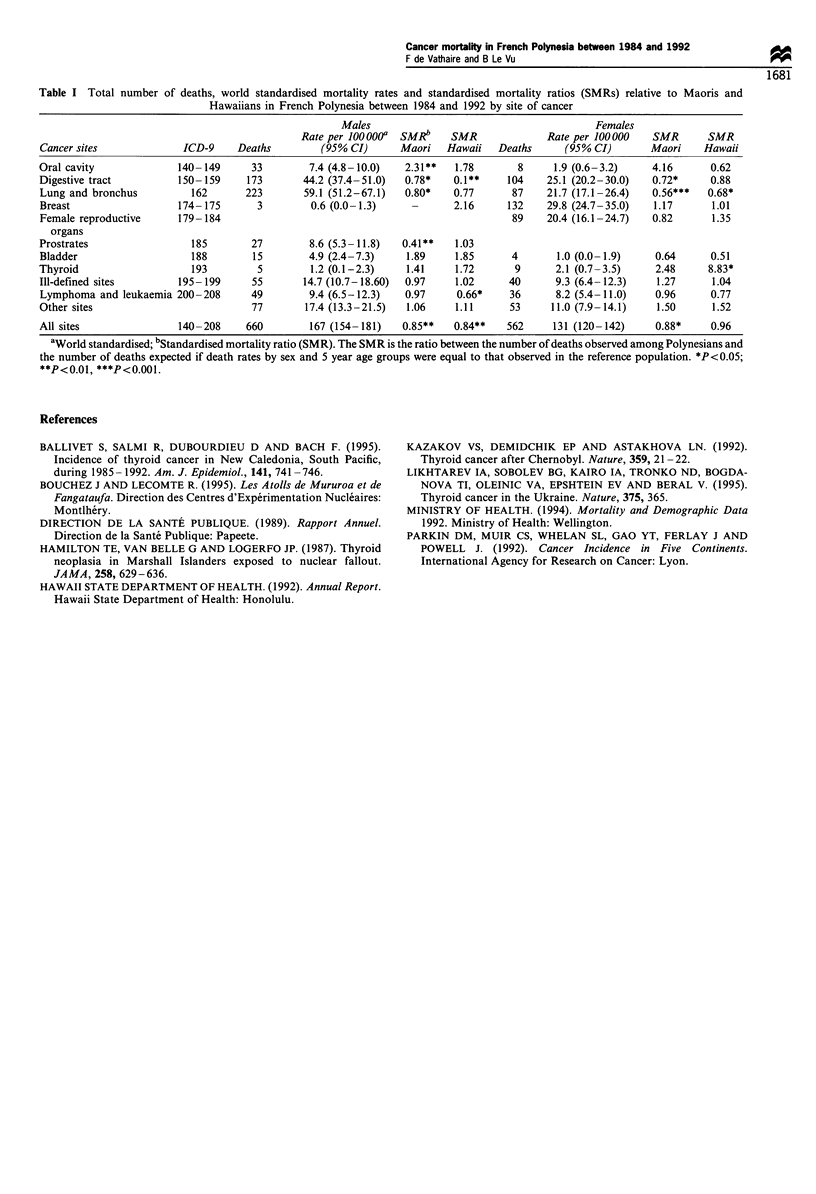# Cancer mortality in French Polynesia between 1984 and 1992.

**DOI:** 10.1038/bjc.1996.609

**Published:** 1996-11

**Authors:** F. de Vathaire, B. Le Vu

**Affiliations:** National Institute of Health and of Medical Research (INSERM), Unit 351, Institut Gustave Roussy, Villejuif, France.

## Abstract

A mortality study of French Polynesia in the period 1984-92, although limited by the small population living close to the test sites and the high proportion of deaths attributed to ill-defined causes, found no excess of cancer that could confidently be attributed to the 41 atmospheric test explosions in 1966-74. A study of cancer incidence is planned.


British Journal of Cancer (1996) 74, 1680–1681
© 1996 Stockton Press All rights reserved 0007–0920/96 $12.00

### Cancer mortality in French Polynesia between 1984 and 1992

F de Vathaire and B Le Vu

*National Institute of Health and of Medical Research (INSERM), Unit 351, Institut Gustave Roussy, rue Camille Desmoulins, 94805 Villejuif, France.*

**Summary** A mortality study of French Polynesia in the period 1984–92, although limited by the small population living close to the test sites and the high proportion of deaths attributed to ill-defined causes, found no excess of cancer that could confidently be attributed to the 41 atmospheric test explosions in 1966–74. A study of cancer incidence is planned.

**Keywords:** nuclear tests; thyroid cancer; epidemiology; geographical study; French Polynesia

The French army carried out 41 atmospheric nuclear test explosions on the atolls of Mururoa and Fangataufa in French Polynesia, between 1966 and 1974 (Bouchez and Lecomte, 1995). At this time no study about cancer mortality in French Polynesia has been published.

French Polynesia is made up of five archipelagos: Iles du Vent, Iles sous le Vent, Marquises, Australes and Tuamotu-Gambier, including about 121 atolls or islands spread over a surface area of 4 million km$^2$. Only 4000 km$^2$ of this area is land. Mururoa and Fangataufa are situated in the Taumotu-Gambier archipelago. French Polynesia is divided into 58 administrative areas called 'communes'. The census of 1988 enumerated 163 790 inhabitants born in French Polynesia, of whom 85% resided in either the Iles du Vent or the Iles sous le Vent, which are more than 1000 km away from Mururoa and Fangataufa. The Tuamotu-Gambier archipelago is the largest and the most sparsely populated archipelago: about 4000 natives are living in a 500 km zone around the two test sites, which are about 100 km apart.

Since January 1984, the cause of each death has been recorded on every island or atoll by a public health employee (Direction de la Santé Publique, 1989). From the Institut Territorial de la Statistique (ITSTAT), we obtained the causes of death in each commune between 1984 and 1992, coded according to the ninth revision of the International Classification of Diseases (ICD-9). We also obtained the census data of 1983 and 1988. We have excluded transient workers from France, by restricting our study to individuals born and living in French Polynesia. Populations at risk in each commune were estimated from the census. We also obtained from IARC (Parkin *et al.*, 1992) the population size and the number of deaths, by site of cancer, for each 5 year age group, during the period 1983 to 1987 in the Maori population of New Zealand, and in the Hawaiian native population. These last two countries are located about 4500 km from Mururoa.

In French Polynesia, 25% of the death certificates from 1984 to 1992 reported a poorly specified or unspecified cause (ICD-9 code: 780–799). This proportion was higher in persons under age 5 (42%) or over age 74 (38%) than at other ages (19%). It also varied widely between archipelagos, from 20% in Iles du Vent to 62% in Tuamotu-Gambier. This variation can be partly explained by the low qualification of the public health staff on the small atolls. Within the Tuamotu-Gambier, this percentage did not vary with the distance from the nuclear sites: 60% in atolls less than 500 km from Mururoa, 72% in those from 500 to 1000 km, and 56% in those more than 1000 km.

For the total population, 1219 deaths from cancer were reported between 1984 and 1992, representing 15% of all deaths. Overall, cancer mortality rates appeared to be higher in the Iles du Vent than in the other archipelagos, but the very high proportion of poorly specified or unspecified cause of death in the other archipelagos represents a major problem in the interpretation of the findings.

Overall, cancer mortality among French Polynesians was 4 to 17% lower than among Maoris and Hawaiians (Table I). These differences were probably explained by the lower rate of poorly specified or unspecified causes of death among Hawaiians [0.8% in 1991 (Hawaii State Department of Health, 1992)] and Maoris [4% in 1992 (Ministry of Health, 1994)].

Thyroid cancer mortality was found to be higher among Polynesians than among Maoris and Hawaiians, whose death rates for 1984–1987 were already among the highest published by IARC (Parkin *et al.*, 1992). However, thyroid cancer incidence is known to be still higher in at least one population not covered by this IARC publication than in Maoris and Hawaiians, namely, another Pacific Island group, New Caledonia, which is located more than 4500 km from Mururoa (Ballivet *et al.*, 1995). The five males and nine females who died from thyroid cancer between 1984 and 1992 resided in the Iles du Vent or in the Iles sous le Vent at time of death, i.e. more than 1000 km from Mururoa. All but one were born on islands located more than 1000 km from Mururoa, and one was born in the Tuamotu-Gambier, about 950 km from Mururoa. They were born between 1902 and 1944 and were, therefore, already aged 20 or more at the time of the first atmospheric test. Based on the experience of the Chernobyl accident in 1986 (Kazakov *et al.*, 1992; Likhtarev *et al.*, 1995) and of the Marshall Islands, which were contaminated by the Bravo nuclear test in 1954 (Hamilton *et al.*, 1987), thyroid cancer caused by massive radioiodine fallout would be expected in individuals irradiated during childhood and residing either near the site or in clusters. Contamination by radioiodine from working in nuclear installations is also an unlikely explanation for our observations, since the excess is observed mainly among women, and very few native women worked on these test sites.

The present analysis fails to show evidence of a generalised excess of cancer mortality in French Polynesia. However, this study is limited by the very small local population living less than 500 km away from the nuclear sites (about 4000 individuals), and the high rate of unknown causes of death. A cancer registry in French Polynesia has been in existence since 1980, and we are planning to analyse these data. Our results show that an incidence study is necessary, which would be more powerful and more precise than the present mortality study.

**Acknowledgements**
We thank Catherine Hill, Monique Lê, Henri Cassagnou and Patrick Ward for their helpful comments, and Jacques Ferlay (IARC) for his collaboration. This work was supported in part by a grant from the Service Mixte de Contrôle Biologique (SMCB).

Correspondence: F de Vathaire
Received May 1996; revised June 1996; accepted June 1996

**Table I** Total number of deaths, world standardised mortality rates and standardised mortality ratios (SMRs) relative to Maoris and Hawaiians in French Polynesia between 1984 and 1992 by site of cancer

| | | Males | | | | Females | | | |
|---|---|---|---|---|---|---|---|---|---|
| *Cancer sites* | *ICD-9* | *Deaths* | *Rate per 100 000[a] (95% CI)* | *SMR[b] Maori* | *SMR Hawaii* | *Deaths* | *Rate per 100 000 (95% CI)* | *SMR Maori* | *SMR Hawaii* |
| Oral cavity | 140–149 | 33 | 7.4 (4.8–10.0) | 2.31** | 1.78 | 8 | 1.9 (0.6–3.2) | 4.16 | 0.62 |
| Digestive tract | 150–159 | 173 | 44.2 (37.4–51.0) | 0.78* | 0.1** | 104 | 25.1 (20.2–30.0) | 0.72* | 0.88 |
| Lung and bronchus | 162 | 223 | 59.1 (51.2–67.1) | 0.80* | 0.77 | 87 | 21.7 (17.1–26.4) | 0.56*** | 0.68* |
| Breast | 174–175 | 3 | 0.6 (0.0–1.3) | – | 2.16 | 132 | 29.8 (24.7–35.0) | 1.17 | 1.01 |
| Female reproductive organs | 179–184 | | | | | 89 | 20.4 (16.1–24.7) | 0.82 | 1.35 |
| Prostrates | 185 | 27 | 8.6 (5.3–11.8) | 0.41** | 1.03 | | | | |
| Bladder | 188 | 15 | 4.9 (2.4–7.3) | 1.89 | 1.85 | 4 | 1.0 (0.0–1.9) | 0.64 | 0.51 |
| Thyroid | 193 | 5 | 1.2 (0.1–2.3) | 1.41 | 1.72 | 9 | 2.1 (0.7–3.5) | 2.48 | 8.83* |
| Ill-defined sites | 195–199 | 55 | 14.7 (10.7–18.60) | 0.97 | 1.02 | 40 | 9.3 (6.4–12.3) | 1.27 | 1.04 |
| Lymphoma and leukaemia | 200–208 | 49 | 9.4 (6.5–12.3) | 0.97 | 0.66* | 36 | 8.2 (5.4–11.0) | 0.96 | 0.77 |
| Other sites | | 77 | 17.4 (13.3–21.5) | 1.06 | 1.11 | 53 | 11.0 (7.9–14.1) | 1.50 | 1.52 |
| All sites | 140–208 | 660 | 167 (154–181) | 0.85** | 0.84** | 562 | 131 (120–142) | 0.88* | 0.96 |

[a]World standardised; [b]Standardised mortality ratio (SMR). The SMR is the ratio between the number of deaths observed among Polynesians and the number of deaths expected if death rates by sex and 5 year age groups were equal to that observed in the reference population. *$P<0.05$; **$P<0.01$, ***$P<0.001$.

### References

BALLIVET S, SALMI R, DUBOURDIEU D AND BACH F. (1995). Incidence of thyroid cancer in New Caledonia, South Pacific, during 1985–1992. *Am. J. Epidemiol.*, **141,** 741–746.

BOUCHEZ J AND LECOMTE R. (1995). *Les Atolls de Mururoa et de Fangataufa.* Direction des Centres d'Expérimentation Nucléaires: Montlhéry.

DIRECTION DE LA SANTÉ PUBLIQUE. (1989). *Rapport Annuel.* Direction de la Santé Publique: Papeete.

HAMILTON TE, VAN BELLE G AND LOGERFO JP. (1987). Thyroid neoplasia in Marshall Islanders exposed to nuclear fallout. *JAMA*, **258,** 629–636.

HAWAII STATE DEPARTMENT OF HEALTH. (1992). *Annual Report.* Hawaii State Department of Health: Honolulu.

KAZAKOV VS, DEMIDCHIK EP AND ASTAKHOVA LN. (1992). Thyroid cancer after Chernobyl. *Nature*, **359,** 21–22.

LIKHTAREV IA, SOBOLEV BG, KAIRO IA, TRONKO ND, BOGDANOVA TI, OLEINIC VA, EPSHTEIN EV AND BERAL V. (1995). Thyroid cancer in the Ukraine. *Nature*, **375,** 365.

MINISTRY OF HEALTH. (1994). *Mortality and Demographic Data 1992.* Ministry of Health: Wellington.

PARKIN DM, MUIR CS, WHELAN SL, GAO YT, FERLAY J AND POWELL J. (1992). *Cancer Incidence in Five Continents.* International Agency for Research on Cancer: Lyon.